# Recurrent acute necrotizing pancreatitis secondary to hypercalcemic crisis in hyperparathyroidism: a case report

**DOI:** 10.1186/s13256-025-05682-5

**Published:** 2025-11-21

**Authors:** Kun Zou, Tianbin Cai, Rui Liu, Junqiang Zhang

**Affiliations:** 1 Intensive Care Unit, The Second Hospital and Clinical Medical School, Lanzhou University, 82 Cuiyingmen, Lanzhou, Gansu Province China; 2 Liuzhou Key Laboratory of Severe Abdominal Infection Research, Liuzhou People’s Hospital, Liuzhou, Guangxi Zhuang Autonomous Region China

**Keywords:** Hypercalcemic crisis, Primary hyperparathyroidism, Recurrent acute pancreatitis, Acute necrotizing pancreatitis, Infected walled-off necrosis, Parathyroid adenoma, Parathyroidectomy, Case report

## Abstract

**Background:**

Although less common, metabolic disorders such as hypercalcemia are implicated in the pathogenesis of acute pancreatitis. Persistent hypercalcemia may lead to recurrent acute pancreatitis if the underlying etiology remains untreated.

**Case presentation:**

A 56-year-old Chinese female patient with a history of hypercalcemia-associated acute pancreatitis 3 years earlier presented with recurrent acute necrotizing pancreatitis lasting for 4 weeks. On admission, she was afebrile with normal inflammatory markers, and computed tomography revealed walled-off pancreatic necrosis without signs of infection, obviating the need for immediate invasive intervention. Laboratory tests showed a hypercalcemic crisis (serum calcium 4.49 mmol/L) and markedly elevated parathyroid hormone (2491 pg/mL). Neck computed tomography and ultrasound identified a left parathyroid adenoma (33 × 21 × 23 mm), implying primary hyperparathyroidism. The patient underwent parathyroidectomy, with histopathology confirming the diagnosis. Postoperatively, serum parathyroid hormone and calcium levels normalized. However, on postoperative day 3, inflammatory markers sharply increased, and repeat abdominal computed tomography detected gas within the necrotic collection, indicating infected necrosis. Necrosectomy and drainage were performed, with cultures identifying *Enterobacter cloacae*. At 3-year follow-up, no recurrence of acute pancreatitis was observed.

**Conclusion:**

Primary hyperparathyroidism should be considered in patients with hypercalcemia, especially those with acute necrotizing pancreatitis. Early diagnosis and parathyroidectomy for primary hyperparathyroidism are critical to preventing hypercalcemia-related complications, including recurrent acute pancreatitis.

## Introduction

Acute pancreatitis (AP) is one of the most common gastrointestinal diseases, with a wide spectrum of etiologies. Gallstones and alcohol abuse constitute the two leading causes, accounting for 21–33% and 16–27% of cases, respectively [1]. Metabolic disorders such as hypercalcemia, although less frequent, are associated with the pathogenesis of AP. Primary hyperparathyroidism (PHPT), a relatively frequent endocrine disease, represents the predominant cause of hypercalcemia, and the reported incidence of AP in PHPT ranges between 1.5% and 13% [2]. The causal relationship between PHPT and pancreatitis, however, has been debated for decades. Some studies, such as a report from the Mayo Clinic by Bess *et al*. [3] and a population-based investigation [2], found no increased risk of AP in patients with PHPT compared with the general population, arguing against a direct causal link. In contrast, other studies [4–6] demonstrated a higher incidence of AP among patients with PHPT, suggesting that PHPT-associated hypercalcemia is a major risk factor for AP. Recurrent AP develops in 10–30% of patients with AP [7] and is defined as more than two discrete episodes of AP with complete resolution for at least 3 months between each episode [8]. Failure to identify and address the underlying etiology predisposes patients to recurrence, underscoring the importance of timely diagnosis and definitive management of PHPT in preventing recurrent AP. We here present a case of recurrent acute necrotizing pancreatitis complicated by infection, in which hypercalcemia secondary to PHPT was identified as the sole etiology. This case highlights the necessity of screening for metabolic causes such as PHPT in patients with recurrent AP.

## Case presentation

**Fig. 1:**
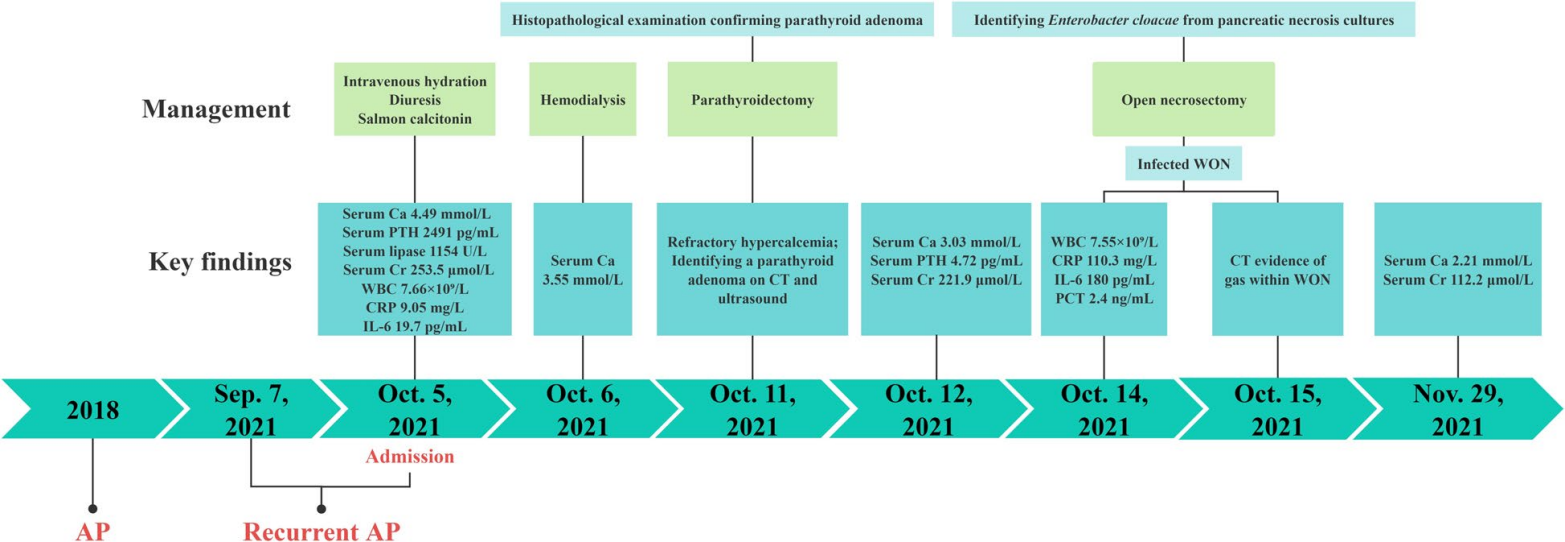
Schematic timeline of diagnostic and therapeutic events. AP, acute pancreatitis; CT, computed tomography; PTH, parathyroid hormone; WON, walled-off necrosis; Ca, calcium; Cr, creatinine; WBC, white blood cell count; CRP, C-reactive protein; IL-6, interleukin 6; PCT, procalcitonin

A 56-year-old Chinese female patient presented to our institution with persistent nausea and vomiting for 5 days, without fever or abdominal pain. She had a history of hypercalcemia-induced AP 3 years earlier. The current admission represents a protracted course following a hospitalization at an outside facility 4 weeks ago for recurrent AP (Fig. 1). During that prior admission, her hospital course was complicated by hypercalcemia and acute kidney injury (AKI), necessitating management with hemodialysis and medications. Additional past medical history included insulin-dependent type 2 diabetes mellitus for 15 years and hypertension treated with nifedipine sustained-release tablets for 10 years. She denied alcohol abuse or biliary disease. Her family history was negative for endocrine disorders and genetic syndromes. She also denied any recent significant psychosocial stressors. Physical examination showed blood pressure of 176/110 mmHg, pulse of 87 beats per minute, respiratory rate of 12 breaths per minute and a temperature of 36.8 °C. There were no stigmata of hyperparathyroidism on examination, such as neck mass or bony tenderness. The abdomen was soft with epigastric tenderness on palpation.

**Fig. 2:**
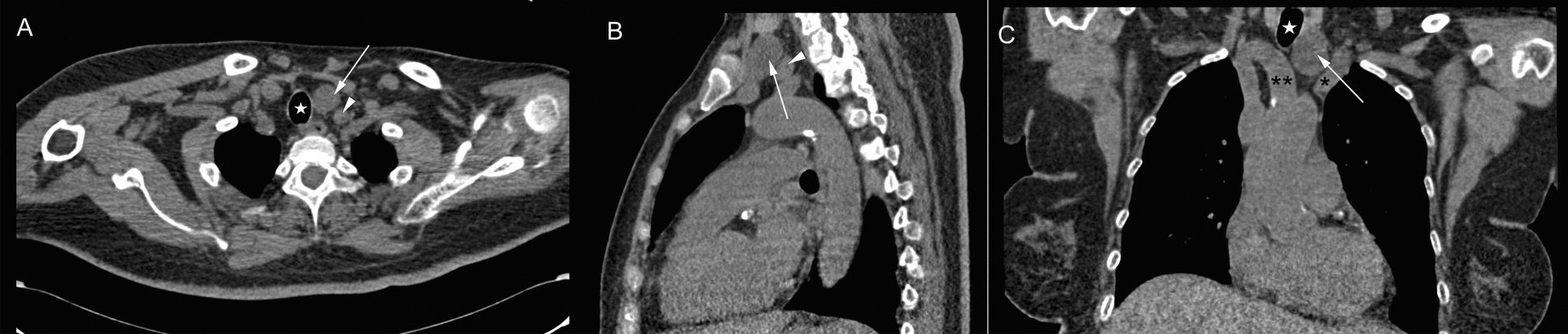
Neck computed tomography scan on admission day. Axial (**A**), sagittal (**B**), and coronal (**C**) computed tomography showing a round hypodense lesion (arrows) between the trachea (stars) and the left carotid artery (arrowheads); asterisk, left brachiocephalic vein; double asterisk, brachiocephalic trunk

**Fig. 3:**
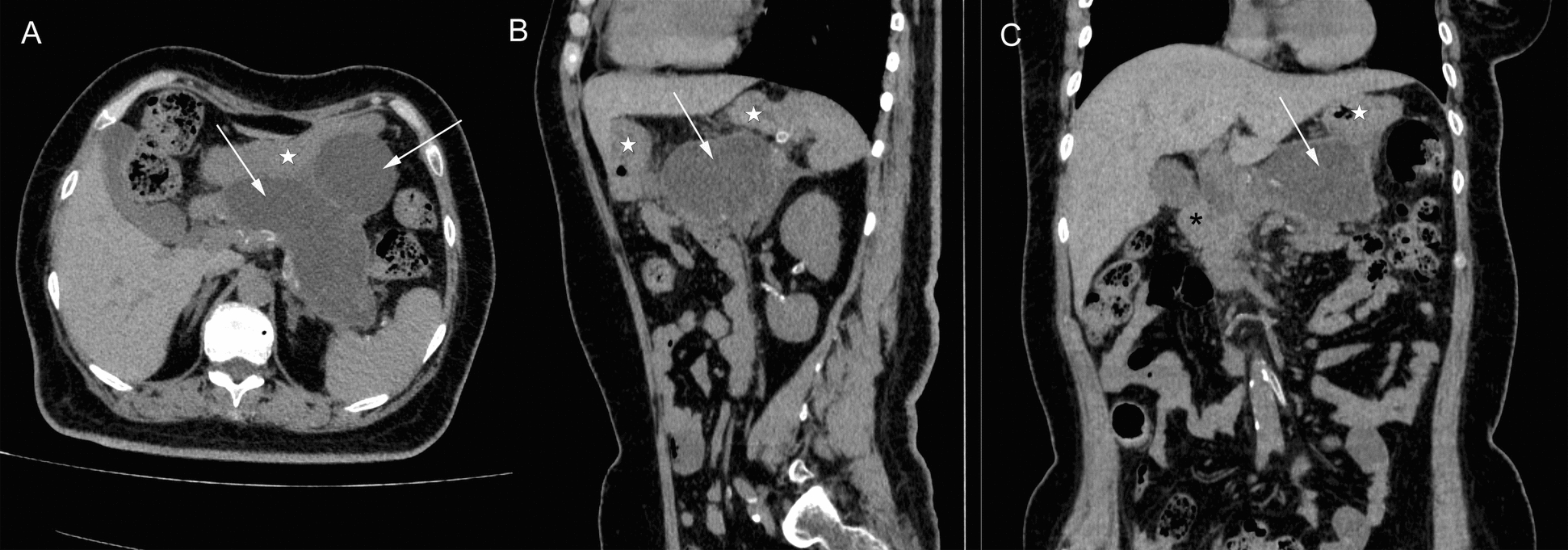
Abdominal computed tomography scan on admission day. Axial (**A**), sagittal (**B**), and coronal (**C**) computed tomography showing a heterogeneous, fully encapsulated necrosis collection in the peripancreatic area (arrows); stars, stomach; asterisk, duodenum

The laboratory tests revealed severe hypercalcemia with serum calcium level of 4.49 mmol/L (reference range 2.11–2.52 mmol/L) and ionized calcium of 2.44 mmol/L (reference range 1.1–1.3 mmol/L). The 24-hour urinary calcium excretion was 9.45 mmol/24 hours (reference range 2.5–7.5 mmol/24 hours). Serum level of intact parathyroid hormone (PTH) increased up to 2491 pg/mL (reference range 10–69 pg/mL), while pituitary hormone profiles were within normal limits. Her serum 25-hydroxy vitamin D was 9 ng/mL (reference range 30–100 ng/mL), serum phosphate 1.19 mmol/L (reference range 0.85–1.51 mmol/L), and serum triglycerides 2.85 mmol/L (reference range 0.56–1.7 mmol/L). Pancreatic enzymes were elevated, including serum lipase 1154 U/L (reference range 23–300 U/L) and serum amylase 203 U/L (reference range 35–135 U/L). Inflammatory parameters were marginally elevated, including C-reactive protein (CRP) 9.05 mg/L (reference range 0–6 mg/L) and interleukin 6 (IL-6) 19.7 pg/mL (reference range 0–7 pg/mL), whereas the white blood cell count was normal. The patient had preexisting renal impairment, with serum creatinine 253.5 μmol/L (reference range 41–73 μmol/L) on admission. Cervical ultrasound showed a well-circumscribed, mixed-echogenic mass (38 mm × 22 mm × 23 mm) posterior to the left thyroid lobe, suggestive of a parathyroid adenoma. Neck computed tomography (CT) confirmed a corresponding round hypodense lesion (33 mm × 21 mm × 23 mm) located between the trachea and the left carotid artery (Fig. 2). Abdominal CT demonstrated a heterogeneous, encapsulated necrosis collection in the peripancreatic area (Fig. 3), in the absence of cholelithiasis or bile duct dilatation.

**Fig. 4:**
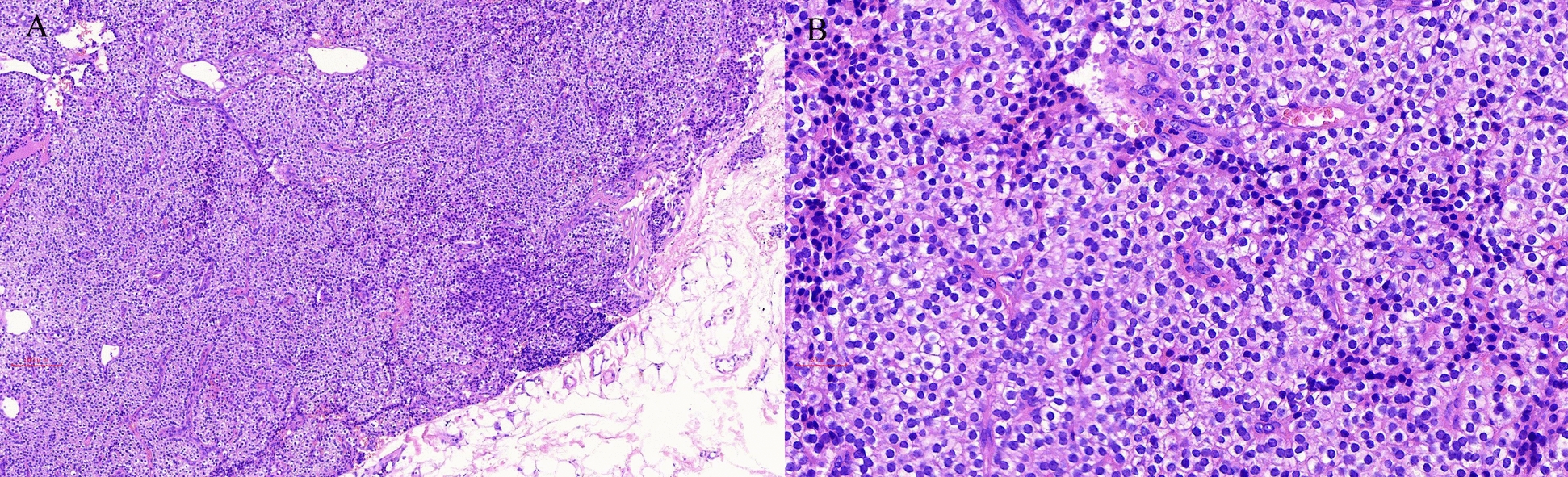
Histopathological examination of parathyroid adenoma. **A** Low-power view (×100) showing a well-encapsulated tumor composed of chief cells arranged in solid nests; **B** high-power view (×400) demonstrating uniform chief cells with round, hyperchromatic nuclei and eosinophilic to clear cytoplasm without significant atypia

On the basis of the clinical, biochemical, and imaging findings, the patient was diagnosed with a hypercalcemic crisis secondary to PHPT, complicated by AKI, recurrent AP, and walled-off necrosis (WON). A complete etiologic workup for AP systematically excluded all common causes, including biliary, alcoholic, and hypertriglyceridemic origins (Table 1). Testing for serum immunoglobulin G4 (IgG4) was omitted as the presentation was not suggestive of autoimmune pancreatitis. The workup identified severe hypercalcemia as the sole prominent abnormality. Given the peripancreatic walled-off necrosis without evidence of infection, invasive intervention was not indicated at this stage. Initial management of the hypercalcemic crisis included aggressive intravenous saline hydration, diuresis with intravenous furosemide, and inhibiting bone resorption with salmon calcitonin. Despite these measures, persistent severe hypercalcemia (serum calcium > 3.5 mmol/L) necessitated emergency hemodialysis. Due to refractory hypercalcemia with post-hemodialysis rebound and confirmed localization of a left parathyroid adenoma, the patient underwent left parathyroidectomy on the sixth hospital day. Histopathological examination confirmed the diagnosis of a parathyroid adenoma (Fig. 4). Postoperatively, serum PTH and calcium levels normalized, though transient hypocalcemia required oral calcium carbonate supplementation.

**Table 1:** Etiologic workup for acute pancreatitis

Etiologic category	Specific tests/findings	Result	Interpretation
Gallstones	Abdominal CT/Ultrasound	No gallstones, no biliary duct dilation	Excluded
Alcohol	Detailed history	Denied abuse	Excluded
Hypertriglyceridemia	Serum triglyceride level	2.85 mmol/L	Unlikely as primary cause
Hypercalcemia	Serum calcium and PTH	Serum calcium 4.49 mmol/L and PTH 2491 pg/mL	Identified
Autoimmune	Clinical presentation; IgG4 (not tested)	No typical features	Less likely
Post-ERCP	Recent procedure history	No relevant history	Excluded
Medication	Medication review	No known pancreatitis-inducing drugs	Less likely
Trauma	Patient history	No history of abdominal trauma	Excluded

CT, computed tomography; PTH, parathyroid hormone; IgG4, immunoglobulin G4; ERCP, endoscopic retrograde cholangiopancreatography

**Fig. 5:**
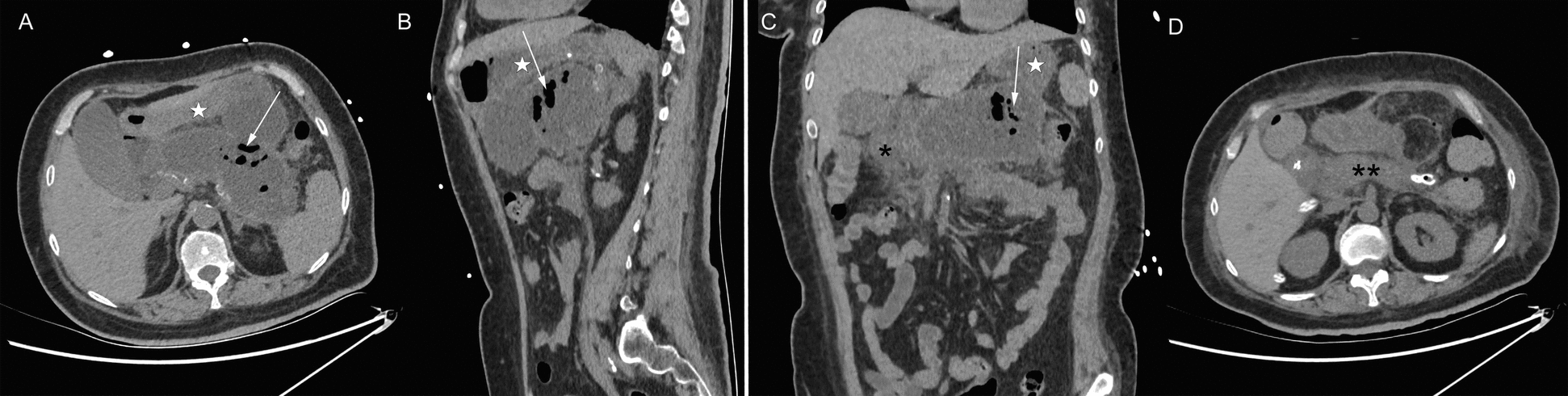
Abdominal computed tomography scan during the perioperative period of the pancreatic necrosectomy. Axial (**A**), sagittal (**B**), and coronal (**C**) computed tomography showing gas formation (arrows) within the walled-off peripancreatic necrotic collection on postoperative day 4 after parathyroidectomy (hospital day 10). **D** resolution of peripancreatic collections on postoperative day 12 after necrosectomy; stars, stomach; asterisk, duodenum; double asterisk, pancreas

On postoperative day 3, the patient complained of epigastric distension, nausea, and vomiting accompanied by markedly elevated inflammatory markers: white blood cell count 10.2 × 10^9^/L, C-reactive protein (CRP) 121.76 mg/L, interleukin (IL)-6 235 pg/mL, and procalcitonin (PCT) 2.4 ng/mL (reference range 0–0.25 ng/mL). Repeat abdominal CT revealed gas formation within the walled-off peripancreatic necrotic collection (Fig. 5A–C), consistent with infected pancreatic necrosis, along with duodenal wall edema. The infection of WON was most likely attributable to the natural history of necrotizing pancreatitis in its late phase, rather than to the prior parathyroidectomy. Due to persistent vomiting, nasojejunal tube feeding was initiated. Empiric antimicrobial therapy with ceftizoxime and ornidazole was administered. Ultrasound-guided percutaneous drainage of the infected pancreatic necrosis was attempted but failed due to anatomical constraints. Given the lack of requisite expertise and equipment for endoscopic drainage or necrosectomy at our institution, open necrosectomy with drainage placement was subsequently performed on day 38 after pancreatitis onset (on postoperative day 4 after parathyroidectomy). Cultures from pancreatic necrotic specimens confirmed *Enterobacter cloacae* infection, and antibiotics were escalated to piperacillin-tazobactam on the basis of antimicrobial susceptibility testing. Given the favorable clinical response, the total duration of antibiotic therapy encompassing both empiric and targeted therapy was 2 weeks. Enteral nutrition via nasojejunal tube resumed on postoperative day 2 after necrosectomy. Temporary nasogastric decompression was required for delayed gastric emptying secondary to duodenal edema. The patient gradually improved and transitioned to oral intake with a normal diet. Follow-up CT demonstrated significant resolution of peripancreatic collections (Fig. 5D). Drainage catheters were removed when the daily drainage output decreased to less than 5 ml for three consecutive days. The patient was subsequently discharged, with follow-up CT confirming complete resolution of the infected necrotizing pancreatitis. She has been followed up for 3 years without recurrence of pancreatitis, and serum levels of PTH and calcium were normal without calcium supplement.

## Discussion

PHPT ranks as the third most prevalent endocrine disorder, with an estimated annual incidence ranging from 0.4 to 82 cases per 100,000 individuals [9]. Women have a three- to fourfold higher predisposition than men [10]. As the predominant etiology of hypercalcemia, PHPT results from abnormal, dysregulated PTH secretion [11], with approximately 80% of cases are caused by solitary parathyroid adenoma [12]. Upon detection of hypercalcemia, a systematic diagnostic evaluation is required to confirm PHPT. Although uncommon, AP represents a severe complication of PHPT. Our patient had experienced AP with hypercalcemia 3 years earlier; however, the underlying etiology of hypercalcemia was not timely identified, ultimately leading to recurrent AP. Therefore, PHPT should be included in the differential diagnosis of all patients presenting with hypercalcemia, particularly those with necrotizing pancreatitis when common causes (for example, gallstones or alcohol abuse) have been excluded. Additionally, multiple endocrine neoplasia type 1 (MEN1) represents a crucial differential diagnosis. It is an autosomal dominant disorder caused by mutations in the *MEN1* gene, typically characterized by combined occurrence of parathyroid tumors, pancreaticoduodenal neuroendocrine tumors, and pituitary adenomas [13]. Our patient had no personal or family history suggestive of MEN1. Aside from PHPT, there were no clinical features of other MEN1-associated tumors, such as symptoms of gastrinoma (for example, peptic ulcer disease) or insulinoma (for example, hypoglycemia). Hormonal evaluation revealed normal anterior pituitary function. Ultimately, a definitive exclusion of MEN1 is limited by the unavailability of genetic testing.

Hypercalcemic crisis is a rare but life-threatening complication of PHPT, which has the potential to cause rapid deterioration of central nervous system, cardiac and renal function if not treated promptly. This condition typically evolves from chronic mild hypercalcemia to acute severe calcium intoxication. While no universally accepted definition exists, hypercalcemic crisis is generally characterized by serum calcium levels exceeding 3.5 mmol/L accompanied by multiorgan dysfunction [14]. As a medical emergency, rapid calcium reduction is critical to prevent fatal complications. First-line therapy involves aggressive intravenous hydration and loop diuretics. However, in PHPT cases refractory to medical management—particularly those with concurrent AKI, as observed in our patient—hemodialysis serves as both an effective salvage therapy and a bridge to parathyroidectomy, the definitive curative intervention. Preoperative hemodialysis to correct hypercalcemia is critical to mitigate intraoperative risks, including life-threatening cardiac arrhythmias.

The causal relationship between PHPT and pancreatitis remains controversial. The 2022 International Workshop guidelines explicitly state that gastrointestinal symptoms (including pancreatitis) are not clearly linked to routine PHPT [15]. Yet multiple series reported higher pancreatitis rates in patients with PHPT than expected, and resolution after surgery [5, 6]. For example, Bai *et al*. reviewed ten retrospective studies, each with > 50 patients, and found that patients with PHPT in eight studies had a higher-than-background pancreatitis rate, and that parathyroidectomy led to resolution of pancreatitis attacks in most cases [16]. In our patient, common causes of AP, including alcohol abuse and cholelithiasis, were absent, while severe hypercalcemia and elevated PTH were evident. Parathyroidectomy normalized calcium levels and prevented AP recurrence over 3 years, suggesting hypercalcemia secondary to PHPT as the likely culprit. The exact mechanism of hypercalcemia-induced AP remains unclear. Gerasimenko *et al*. [17] demonstrated that sustained cytosolic calcium elevation triggers premature pro-enzyme activation in acinar cells.

AP is morphologically classified into interstitial edematous pancreatitis and necrotizing pancreatitis (NP). In contrast to the former, NP may progress to WON in its late phase (typically after 4 weeks), which develops a well-defined capsule enclosing liquid and solid necrotic debris. For the management of NP, we followed the principles of international pancreatitis guidelines [18, 19], such as early enteral nutrition, judicious antibiotic use, and delayed invasive intervention. Infected WON carries high morbidity and mortality, and a step-up approach [19] including percutaneous drainage or endoscopic transluminal drainage, minimally invasive necrosectomy, and open necrosectomy is currently recommended as the primary treatment strategy. Percutaneous or endoscopic drainage alone is adequate in 30–50% of patients with WON [20]. If drainage fails, endoscopic necrosectomy should be preferred for infected WON. However, the choice between endoscopic and surgical approaches requires careful individualization. A multicenter randomized trial [21] demonstrated that the endoscopic step-up approach was not superior to the surgical step-up approach in reducing the primary composite endpoint of major complications or mortality in patients with infected NP. Additionally, over-rigid emphasis on the step-up approach without attention to individualization leads to missed optimal surgical timing or even death [22]. Pancreatic necrotic tissue includes completely liquefied necrotic tissue and solid or semisolid necrosis. Therefore, in patients with extensive solid necrosis or where technical barriers preclude endoscopic access (as in our case), open necrosectomy remains a critical therapeutic option [23]. CT density could quantify the proportion of solid necrotic material and is a significant predictor of surgery [24, 25]. A higher mean CT density in infected WON indicates a greater proportion of solid necrosis and a higher likelihood of requiring surgical necrosectomy [26].

Several limitations should be acknowledged. Firstly, the exclusion of MEN1 was based solely on clinical evaluation due to the unavailability of genetic testing. Secondly, the management of infected WON was constrained by local technical resources, necessitating an open necrosectomy rather than a potentially less invasive endoscopic approach.

## Conclusion

Hypercalcemic crisis is a rare but life-threatening complication of PHPT; rapidly decreasing serum calcium could effectively reduce the risk of fatal complications. Hypercalcemia caused by PHPT is a recognized risk factor for AP, highlighting the importance of timely diagnosis and treatment of PHPT to prevent recurrent AP. PHPT should be considered in patients presenting with hypercalcemia, particularly when hypercalcemia occurs in the context of acute necrotizing pancreatitis.

## Key clinical messages

- Screen for PHPT in patients with recurrent AP, particularly when common etiologies are absent and hypercalcemia is present.
- Treat hypercalcemic crisis (> 3.5 mmol/L) as a medical emergency, requiring prompt intravenous hydration, diuresis, and consideration of hemodialysis.
- Perform parathyroidectomy as the definitive curative treatment for PHPT to normalize calcium and prevent recurrent AP.
- Individualize the management of infected NP; open necrosectomy is a critical option when endoscopic techniques are unavailable or solid necrosis predominates.

## Data Availability

Not applicable.
